# Corticosteroid Weaning in Stable Heart Transplant Patients: Guidance by Serum Cortisol Level

**DOI:** 10.1155/2018/3740395

**Published:** 2018-02-18

**Authors:** David A. Baran, Cheryl Rosenfeld, Mark J. Zucker

**Affiliations:** ^1^Advanced Heart Failure and Transplant, Sentara Heart Hospital, Norfolk, VA, USA; ^2^Heart Failure Treatment and Transplant Program, Newark Beth Israel Medical Center, Newark, NJ, USA

## Abstract

**Background:**

Despite earlier studies describing the feasibility of steroid weaning in heart transplant patients, the majority of patients are maintained on steroid therapy for life. We examined a strategy based on a single morning serum cortisol measurement.

**Methods:**

We assigned stable posttransplant patients, who were maintained on tacrolimus, mycophenolate mofetil, and corticosteroids, into one of two groups based on a screening morning cortisol level. Patients with a cortisol < 8 micrograms/deciliter were assigned to a “maintenance” group and the others were assigned to the weaning group and steroids were tapered off over 4–6 weeks. Patients were monitored on subsequent office visits for adrenal insufficiency and allograft rejection.

**Results:**

Thirty-one patients were enrolled (6 patients in the maintenance group and 25 in the steroid-weaning group). Mean follow-up was 10.2 ± 4 years for the weaning group and 9.0 ± 4.9 years in the maintenance group (*p* = 0.6). No cases of rejection were noted, nor did any patient resume steroid treatment following discontinuation.

**Conclusion:**

Steroids can be safely discontinued in stable heart transplant patients with an AM serum cortisol ≥ 8 micrograms/deciliter with appropriate outpatient follow-up. In this study, no patient suffered late rejection or clinically noted adrenal insufficiency.

## 1. Introduction

Corticosteroids have been an integral part of immunosuppression regimens since the beginnings of clinical transplantation [1, 2]. The first heart transplant patients were treated with corticosteroids and antiproliferative medications such as azathioprine. With the addition of cyclosporine A to corticosteroids and antiproliferative drugs [3], heart transplantation changed from a medical experiment to a viable approach to the treatment of end-stage heart failure [4, 5]. The complications of long-term steroid use are well known. While some early studies described the feasibility of steroid weaning [6–10], the idea has not been embraced, and International Society for Heart and Lung Transplantation (ISHLT) registry data indicates that more than 60% of patients are chronically maintained on corticosteroids [11]. The fear of allograft rejection and the concern about “unmasking” occult adrenal insufficiency are the main clinical reasons that more patients are not routinely discontinued from steroid therapy [7, 12, 13].

In the setting of tacrolimus-based immunosuppression, others have reported success with steroid-weaning posttransplantation [14–16]. The ISHLT Guidelines for the Care of Heart Transplant Recipients indicate that corticosteroid weaning is reasonable, especially in patients with significant side effects [17]. However, there are no data in heart transplant recipients to predict which patients will manifest clinical signs of adrenal insufficiency due to chronic steroid therapy.

Administration of corticosteroids suppresses the hypothalamic-pituitary-adrenal (HPA) axis, and ways to assess this include the following: morning serum cortisol level, synthetic adrenocorticotropic hormone (ACTH) stimulation test, ovine corticotrophin releasing hormone (CRH) stimulation test, and insulin-induced hypoglycemia [18]. While both CRH and cortisol levels may be more reliable than ACTH testing [19, 20], an attempt at identifying a specific threshold for steroid withdrawal has been made only with serum cortisol level [19]. Use of the ACTH stimulation test has been reported in kidney transplant patients, but this test requires multiple blood draws and synthetic ACTH is expensive [21]. Using the serum cortisol test to guide withdrawal of corticosteroids in heart transplant recipients has not been reported previously.

It was hypothesized that a single morning cortisol measurement could identify stable heart transplant recipients who could be successfully removed from steroids.

## 2. Methods

Adult heart transplant recipients who were chronically maintained on tacrolimus, mycophenolate mofetil, and corticosteroids and were at least 4 months after transplant were eligible for study enrollment. Patients were recruited from April 2004 to February 2005. All patients were chronically receiving prednisone at a dose between 2.5 and 10 milligrams daily. Exclusion criteria included recipients of a second transplant, combined heart-kidney transplants, treated allograft rejection in the preceding six months, or prior evidence of adrenal failure. Patients who were receiving cyclosporine-based therapy were excluded as well. The study protocol was approved by the local Institutional Review Board.

After informed consent, subjects had a comprehensive metabolic panel including serum sodium, potassium, blood urea nitrogen, and creatinine. In addition, a serum cortisol level was drawn prior to 9 AM.

We arbitrarily picked a threshold value of 8 micrograms/deciliter based on prior publications and clinical experience [18, 19]. There are no studies which have used this approach in the past, with most reported work utilizing a cortisol stimulation test.

**Figure 1:**
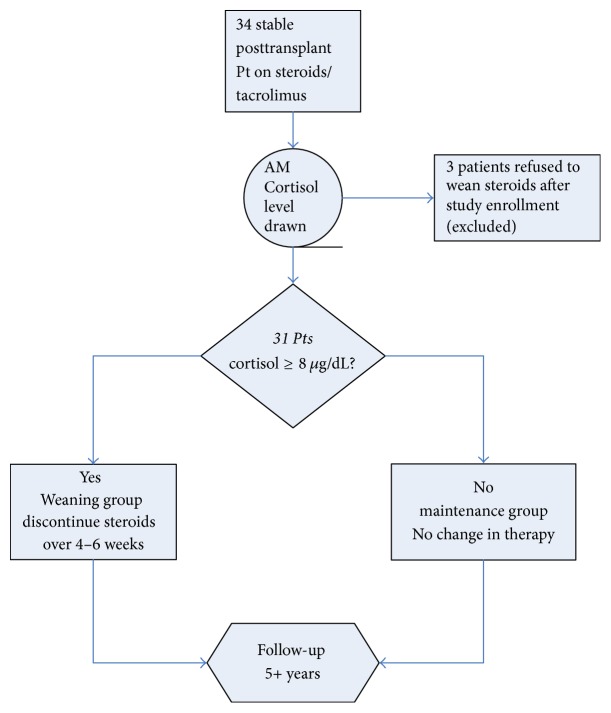
Study flow diagram.

If a patient's serum cortisol was less than 8 micrograms/deciliter then the patient was assigned to the “maintenance” group, and corticosteroid dosage was maintained. The serum cortisol was repeated if the value was close to the cut point. For those patients with cortisol levels at or above 8 micrograms/deciliter, steroids were weaned to discontinuation over 4–6 weeks as an outpatient. Three patients refused to discontinue steroids, despite adequate serum cortisol values, and were excluded from further analysis.  Figure 1 shows the study enrollment graphically.

After study entry, patients were monitored at each visit for signs of adrenal insufficiency (overt fatigue, muscle aches, dizziness, or syncope). We did not employ a symptom questionnaire or routinely check orthostatic blood pressure measurements. In addition, the patients underwent standard posttransplant screening including biopsies, serum sodium, calcium, phosphorus, and magnesium as indicated. Cardiac biopsies were performed at least once after complete steroid discontinuation.

The aims of the study were to validate the use of morning serum cortisol level as a simple test for hypothalamic pituitary-adrenal axis competency, as well as examining the clinical outcomes following steroid weaning including the risk of late rejection and death.

## 3. Results

From April 2004 to February 2005, 34 eligible adult heart transplant patients were enrolled. Subsequently, 3 patients declined to wean corticosteroids regardless of the protocol and they were excluded from further analysis. Six patients had a morning serum cortisol less than 8 micrograms/deciliter and were assigned to the maintenance group, and 25 patients were assigned to the steroid-weaning group.

The baseline demographics of the study groups are in Table 1. There were no significant differences between the group characteristics. Mean follow-up from date of study entry to death or July 2017 was 10.2 ± 4 years for the weaning group and 9.0 ± 4.9 years in the maintenance group (*p* = 0.61, *t*-test).

**Figure 2:**
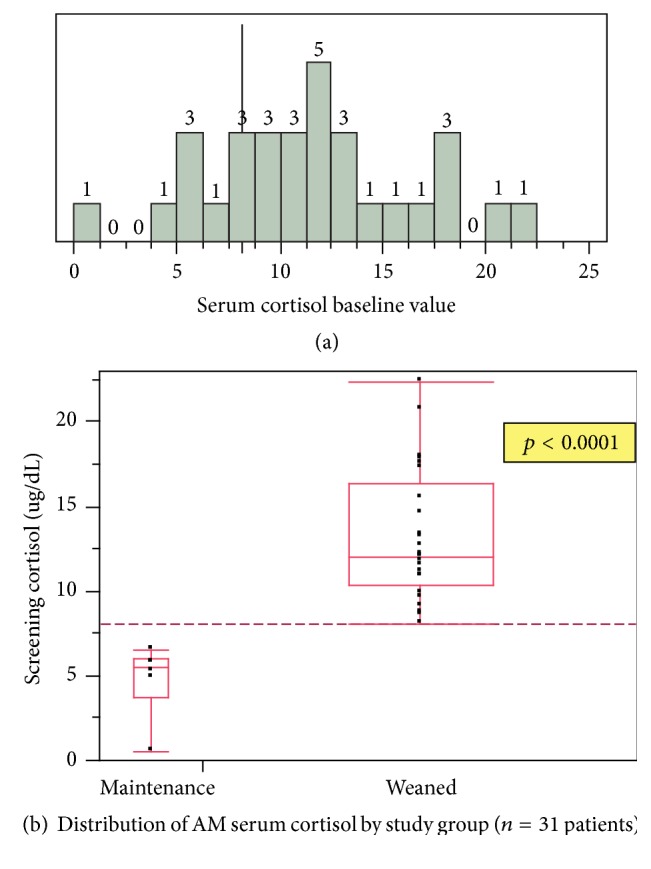


Figure 2(a) shows the distribution of morning cortisol measurements in this study. The median cortisol in the weaning group was 12 (IQR 10.4–16.3)*μ*g/dL and 5.55 (IQR 3.7–6.0) for the patients maintained on long-term corticosteroids (*p* < 0.0001). The mean cortisol was 11.5 ± 4.9 with the 95% confidence intervals at 9.7–13.3*μ*g/dL. The vertical line shows the prospectively chosen level of 8 mcg/dL which appears to be an appropriate cut point to distinguish between groups.  Figure 2(b) shows the distribution of baseline cortisol values between groups demonstrating little overlap between groups.

Patients had at least one endomyocardial biopsy in the first 2 months following steroid discontinuation. There were no cases of rejection (ISHLT grade 2R or higher) noted, and no patient resumed steroid therapy following discontinuation. No episodes of antibody mediated rejection or unexplained graft dysfunction were seen in this cohort of patients.

**Figure 3:**
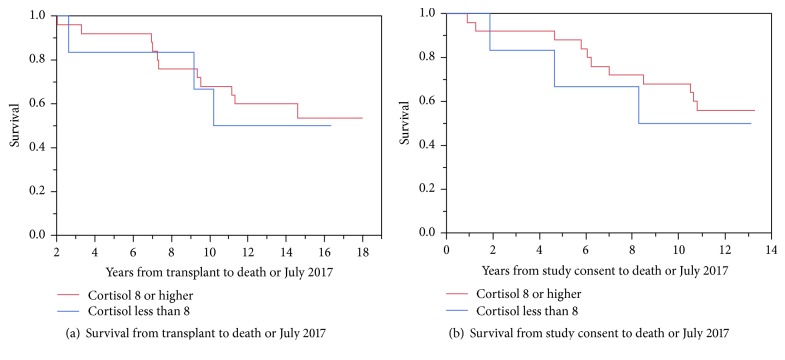
Survival after study enrollment.

Survival after study enrollment was examined and is illustrated in Figure 3. In Figure 3(a), the survival of the cohort from the date of transplantation to death or July 2017 is depicted.  Figure 3(b) shows survival beginning on the day of study consent, ending in death or July 2017 which represents approximately 10 years of follow-up. The survival of the steroid-weaning group was not significantly different from that of the corticosteroid maintenance group, but the analysis is hampered by a small sample size (grossly underpowered for mortality).

## 4. Discussion

This is the first prospective trial of corticosteroid weaning in stable heart transplant patients guided by a laboratory measurement. The widely available serum cortisol measurement facilitated selection of patients where steroid discontinuation was well tolerated. Importantly, no patient suffered allograft rejection, nor graft dysfunction (such as might be seen with antibody mediated rejection). These results are strengthened by an average follow-up of approximately 10 years. Patients were screened for symptoms of adrenal insufficiency at clinic follow-up visits and none were noted. Long-term follow-up supports the efficacy and safety of this approach.

The choice of 8 ng/dL was made based on best judgement and the scant literature which exists. The distribution of the cortisol values at study entry shows that more than 95% of patients were above this value, but it is not possible to know that those with low cortisol could not be successfully weaned from corticosteroids, since such patients were excluded from a weaning attempt. Nevertheless, the approach described in this paper would be applicable to the vast majority of patients in routine clinical practice.

Most of the patients in the trial were on 5 mg at baseline, and typically the dose was reduced to zero over a 4–6-week period by tapering weekly to 1 mg daily and then 1 mg every other day and so forth until the patients were free of corticosteroids. If there was subclinical hypoadrenalism, this approach would allow gradual improvement of native adrenal function if possible.

Corticosteroid discontinuation is not a new idea in transplantation, with reports beginning shortly after the introduction of cyclosporine [8, 16, 22–24]. However, most reports demonstrate only moderate success with corticosteroid discontinuation with 25–40% of patients maintained on corticosteroids long term [25–27]. Most of these studies have aimed to discontinue steroids over the first year after transplant, and several have utilized induction antibody preparations to “facilitate” steroid weaning.

There have been several prospective studies of steroid weaning early after transplantation. Mehra described outcomes for 41 patients treated with tacrolimus and mycophenolate mofetil. 62% of these patients were withdrawn from steroids by an average of 20 ± 11 months after transplant. Meiser et al. reported on 33 patients treated with tacrolimus and sirolimus and all were successfully discontinued from steroids by 6 months after transplant [28]. Yamani et al. published the results for a prospective trial of 32 heart transplant recipients who were randomized to thymoglobulin, tacrolimus, and mycophenolate mofetil without any maintenance corticosteroids, versus a single dose of thymoglobulin followed by conventional triple therapy with tacrolimus, mycophenolate mofetil, and chronic corticosteroids [29]. One of the twelve patients (8%) in the steroid-free group required long-term steroids due to resistant rejection.

Keogh et al. reported on a prospective trial of cyclosporine/azathioprine and steroids versus cyclosporine and azathioprine without steroids in 1992 [7]. Approximately half of the steroid-free patients required institution of chronic steroids due to rejection however. More recently, Crespo-Leiro et al. reported on 24 stable heart transplant recipients who had not had any acute allograft rejection for at least 4 years and were weaned from corticosteroids in a study [12]. Of concern, 25% of the patients (6/24) developed ISHLT grade 2R or greater rejection which necessitated treatment with intravenous steroids, although no episode was fatal. 75% of the patients were on cyclosporine-based regimens with the remainder on tacrolimus-based therapy. Castel et al. reported results for 86 patients who were stable after 24 ± 12 months after transplant [25]. They were able to discontinue steroids in 63% of the patients, with the most common reason for failure being acute cellular rejection. Of note, 64% of the patients were on tacrolimus but the authors did not correlate drug regimen to clinical outcomes.

One key difference between the current study and those in the past is that all patients in this study were receiving tacrolimus as part of the study entry criteria. Perhaps owing to higher antirejection potency [30], none of the patients in this study required resumption of corticosteroid therapy with an extended follow-up interval. The study is obviously not powered to assess mortality, but the lack of a negative trend for the weaning group is encouraging.

The other major result from this study is the utility of the AM serum cortisol to assess the hypothalamic-pituitary access in a simple and effective way. None of the patients with an AM cortisol of at least 8 ng/dL developed clinical adrenal insufficiency or required a return to corticosteroid therapy. However, we cannot comment on the patients with a low cortisol since they were not “challenged” by attempting steroid withdrawal. In addition, the serum cortisol would not be expected to have any influence on the patient's propensity to experience allograft rejection. The data support the safety of discontinuing steroids for patients with an AM cortisol value of at least 8 ng/dL and the distribution of the cortisol measurements suggests that this is a good discriminator with very few patients near the cut point.

The benefits of steroid discontinuation have been documented by others and were not systematically assessed in this study [16, 31, 32]. The number of patients is small and bone density tests were not universally available. Therefore, the clinician will need to balance the risks and benefits for each specific patient but the current study suggests that discontinuation of steroids is feasible and does not result in clinical adrenal insufficiency in patients screened with an AM cortisol measurement. The population is a selected group and this was not a randomized study.

## 5. Conclusion

This is the first prospective trial of corticosteroid weaning guided by a single AM serum cortisol measurement in stable heart transplant recipients. Steroid weaning in patients with a serum cortisol ≥ 8*μ*g/dL was well tolerated with no rejection or return to corticosteroid therapy over the follow-up period.

## Figures and Tables

**Table 1:** Baseline demographics.

Demographics	Maintenance group (*n* = 6)	Weaning group (*n* = 25)	*p* value
Male, *n* (%)	5 (83%)	21 (84%)	0.97
Female, *n* (%)	1 (16.7%)	4 (16%)	0.97
Mean age at transplant	54.5 ± 11.3 years (37–69)	55 ± 10 years (38–69)	0.92
Mean baseline prednisone dose	5.8 ± 2.5 mg (2.5–10)	4.6 ± 1.5 mg (2.5–10)	0.13
Baseline cortisol level (*µ*g/dL)	4.8 ± 2.2 (0.5–6.5)	13.2 ± 3.9 (8.1–22.4)	<0.0001
Baseline creatinine (mg/dL)	1.4 ± 0.2 (1.2–1.8)	1.4 ± 0.4 (0.8–2.2)	0.9
Time after transplant to study entry	2.2 ± 2.0 years (0.3–5.5)	1.9 ± 1.5 years (0.3–4.9)	0.68
Ischemic cardiomyopathy *n* (%)	3 (50%)	13 (52%)	0.93
African American *n* (%)	2 (33%)	7 (28%)	0.99
Pretransplant diabetes mellitus *n* (%)	2 (33%)	3 (12%)	0.24
History of smoking *n* (%)	3 (50%)	16 (64%)	0.78
Statin use (after transplant)	6 (100%)	25 (100%)	NS

## Abbreviations

ISHLT: International Society for Heart and Lung Transplantation
ACTH: Adrenocorticotropic hormone
IQR: Intraquartile range.

## References

[1] Bell P. R. F., Calman K. C., Wood R. F. M. (1971). Reversal of acute clinical and experimental organ rejection using large doses of intravenous prednisolone. *The Lancet*.

[2] Lower R. R. (1969). Rejection of the transplanted heart. *Transplantation Proceedings*.

[3] Devineni R., McKenzie N., Keown P., Kostuk W., Stiller C., Silver M. (1984). Cyclosporine in cardiac transplantation. *Canadian Journal of Surgery*.

[4] Cohen D. J., Loertscher R., Rubin M. F., Tilney N. L., Carpenter C. B., Strom T. B. (1984). Cyclosporine: A new immunosuppressive agent for organ transplantation. *Annals of Internal Medicine*.

[5] Barnhart G. R., Goldman M. H., Hastillo A. (1984). Comparison of immunosuppression therapy following heart transplantation: pretransfusion/azathioprine/atg/prednisone versus cyclosporine/prednisone. *Journal of Heart Transplantation*.

[6] Bolman 3rd. R. M., Olivari M. T., Saffitz J. (1987). Current results with triple therapy for heart transplantation.. *Transplantation Proceedings*.

[7] Keogh A., Macdonald P., Harvison A., Richens D., Mundy J., Spratt P. (1992). Initial steroid-free versus steroid-based maintenance therapy and steroid withdrawal after heart transplantation: Two views of the steroid question. *The Journal of Heart and Lung Transplantation*.

[8] Olivari M.-T., Jessen M. E., Baldwin B. J. (1995). Triple-drug immunosuppression with steroid discontinuation by six months after heart transplantation. *The Journal of Heart and Lung Transplantation*.

[9] Price G. D., Olsen S. L., Taylor D. O., O'Connell J. B., Bristow M. R., Renlund D. G. (1992). Corticosteroid-free maintenance immunosuppression after heart transplantation: Feasibility and beneficial effects. *The Journal of Heart and Lung Transplantation*.

[10] Seydoux C., Berguer D. G., Stumpe F. (1997). Does early steroid withdrawal influence rejection and infection episodes during the first 2 years after heart transplantation?. *Transplantation Proceedings*.

[11] Stehlik J., Edwards L. B., Kucheryavaya A. Y. (2010). The Registry of the International Society for Heart and Lung Transplantation: Twenty-seventh official adult heart transplant report—2010. *The Journal of Heart and Lung Transplantation*.

[12] Crespo-Leiro M. G., Paniagua M. J., Franco R. (2007). Late Steroid Withdrawal After Heart Transplantation and Incidence of Acute Rejection. *Transplantation Proceedings*.

[13] Rosenbaum D. H., Adams B. C., Mitchell J. D., et al. (2006). Effects of early steroid withdrawal after heart transplantation. *The Annals of Thoracic Surgery*.

[14] Baran D. A., Galin I. D., Segura L. (2002). Tacrolimus and cardiac transplantation: A comparison of monotherapy and steroid-dependent patients. *Transplantation Proceedings*.

[15] Baran D. A., Segura L., Kushwaha S. (2001). Tacrolimus monotherapy in adult cardiac transplant recipients: Intermediate-term results. *The Journal of Heart and Lung Transplantation*.

[16] Mehra M. R., Uber P. A., Park M. H., Ventura H. O., Scott R. L. (2004). Corticosteroid weaning in the tacrolimus and mycophenolate era in heart transplantation: Clinical neurohormonal benefits. *Transplantation Proceedings*.

[17] Dipchand A., McCrindle B., West L. (2003). Mycophenolate mofetil: clinical applications and pharmacokinetic monitoring in pediatric heart transplant recipients. *The Journal of Heart and Lung Transplantation*.

[18] Richter B., Neises G., Clar C. (2002). Glucocorticoid withdrawal schemes in chronic medical disorders. *Endocrinology and Metabolism Clinics of North America*.

[19] Courtney C. H., McAllister A. S., McCance D. R. (2000). Comparison of one week 0900 h serum cortisol, low and standard dose synacthen tests with a 4 to 6 week insulin hypoglycaemia test after pituitary surgery in assessing HPA axis. *Clinical Endocrinology*.

[20] Gellner R., Stange M., Schiemann U., Domschke W., Hengst K. (1999). CRH test prior to discontinuation of long-term low-dose glucocorticoid therapy. *Experimental and Clinical Endocrinology & Diabetes*.

[21] Baz-Hecht M., Osher E., Yachnin T. (2006). The low-dose (1 *μ*g) adrenocorticotropin stimulation test in kidney and kidney-pancreas transplant patients: A potential guideline for steroid withdrawal. *Clinical Transplantation*.

[22] Felkel T. O., Smith A. L., Reichenspurner H. C. (2002). Survival and incidence of acute rejection in heart transplant recipients undergoing successful withdrawal from steroid therapy. *The Journal of Heart and Lung Transplantation*.

[23] Kobashigawa J. A., Stevenson L. W., Brownfield E. D., et al. (1992). Initial success of steroid weaning late after heart transplantation. *The Journal of Heart and Lung Transplantation*.

[24] Yacoub M., Alivizatos P., Khaghani A., Mitchell A. (1985). The use of cyclosporine, azathioprine, and antithymocyte globulin with or without low-dose steroids for immunosuppression of cardiac transplant patients. *Transplantation Proceedings*.

[25] Castel M. A., Vallejos I., Ramos P. (2009). Outcome After Steroid Withdrawal in Heart Transplantation. *Transplantation Proceedings*.

[26] Crespo-Leiro M., Delgado J., Almenar L. (2009). Steroid Use in Heart Transplant Patients in Spain in the Current Era: A Multicenter Survey. *Transplantation Proceedings*.

[27] Singh T. P., Faber C., Blume E. D. (2010). Safety and early outcomes using a corticosteroid-avoidance immunosuppression protocol in pediatric heart transplant recipients. *The Journal of Heart and Lung Transplantation*.

[28] Meiser B., Kaczmarek I., Mueller M. (2007). Low-dose Tacrolimus/Sirolimus and Steroid Withdrawal in Heart Recipients Is Highly Efficacious. *The Journal of Heart and Lung Transplantation*.

[29] Yamani M. H., Taylor D. O., Czerr J. (2008). Thymoglobulin induction and steroid avoidance in cardiac transplantation: Results of a prospective, randomized, controlled study. *Clinical Transplantation*.

[30] Scott L. J., McKeage K., Keam S. J., Plosker G. L. (2003). Tacrolimus: a further update of its use in the management of organ transplantation. *Drugs*.

[31] Baran D. A., Ashkar J., Galin I. D. (2002). Tacrolimus and new onset diabetes mellitus: The effect of steroid weaning. *Transplantation Proceedings*.

[32] Lubitz S. A., Baran D. A., Alwarshetty M. M. (2006). Improved Survival With Statins, Angiotensin Receptor Blockers, and Steroid Weaning After Heart Transplantation. *Transplantation Proceedings*.

